# Design of a feature-optimized hybrid CNN–Transformer model for melanoma-vs.-non-melanoma classification

**DOI:** 10.3389/frai.2026.1936676

**Published:** 2026-10-29

**Authors:** R. Ramya, N. Jaisankar

**Affiliations:** School of Computer Science and Engineering (SCOPE), Vellore Institute of Technology (VIT), Vellore, India

**Keywords:** attention fusion, boundary-aware learning, CNN–Transformer, dermoscopy, Grad-CAM++, melanoma classification, Swin Transformer

## Abstract

**Introduction:**

Accurate melanoma recognition from dermoscopic images is challenging because lesion appearance varies substantially across acquisition devices, skin backgrounds, and diagnostic categories.

**Methods:**

This study presents Lesion-aware Hybrid CNN-Transformer Network (LHCT-Net), a lesion-aware hybrid CNN-Transformer framework for binary melanoma-vs.-non-melanoma classification on the 25,331-image ISIC 2019 training collection. The endpoint is defined explicitly as melanoma (positive class) vs. all remaining ISIC 2019 diagnostic categories (negative class); consequently, the reported results are not interpreted as benign-vs.-malignant or general skin-cancer detection performance. The pipeline combines fixed U-Net-based lesion localization, training-only mask-guided lesion augmentation, a boundary-aware EfficientNet-B0 branch for local morphology, a Swin Transformer branch for global context, attention-based local-global fusion, a Lightweight Feature Optimization Block (LFOB), and qualitative Grad-CAM++ visualization. The dataset was evaluated using a fixed 70:15:15 stratified image-level holdout split, with augmentation and hyperparameter selection restricted to the training and validation partitions and the test partition used only for final evaluation.

**Results:**

On the locked test set, the confusion matrix contained 3,096 true negatives, 26 false positives, 29 false negatives, and 650 true positives, corresponding to 98.55% accuracy, 96.15% melanoma precision, 95.73% melanoma sensitivity, 99.17% specificity, 95.94% F1-score, 97.45% balanced accuracy, and an MCC of 0.951. The reported ROC-AUC was 0.98.

**Discussion:**

These results indicate strong discrimination for the defined melanoma-vs.-non-melanoma task, while external validation, subgroup analysis, calibration, and expert validation of explanations remain necessary before clinical use.

## 1 Introduction

One of the most common cancers in the world is skin cancer that results from the unrestricted division of irregular skin cells because of constant exposure to UltraViolet (UV) radiation. Skin cancers are generally divided into three categories: Basal Cell Carcinoma (BCC), Squamous Cell Carcinoma (SCC), and melanoma. Among the various skin cancers, melanoma is considered more severe than BCC and SCC because it is highly metastatic. It has been reported globally that skin cancers are increasing rapidly and are causing great public health challenges (Esteva et al., 2017; Haenssle et al., 2018). Timely clinical recognition of melanoma is important because prognosis worsens after metastatic spread. Dermoscopic assessment can nevertheless be challenging because lesion appearance varies across patients, acquisition devices, and diagnostic categories.

The rising cases of skin cancers have brought to light the importance of effective and automated ways to diagnose such diseases. Access to specialized dermatological assessment is uneven, motivating research on automated decision-support methods for dermoscopic images. Conventional assessment depends on clinical expertise and may be difficult to scale in settings with limited specialist access. Moreover, because benign lesions look almost like malignant ones, confusion may arise even with a medical practitioner. These factors make it vital to utilize intelligent computing tools for diagnosing skin cancers (Tschandl et al., 2020).

In the recent past, Deep Learning (DL) has emerged as an efficient technique for automatic skin cancer detection and classification. Convolutional Neural Networks have shown great potential in extracting hierarchical features of lesions like texture, color distribution, and boundary information with accuracy close to that achieved by dermatologists in classification (Esteva et al., 2017). Several studies have attempted to explore more sophisticated techniques in order to increase the diagnostic efficiency of DL models.

Akinrinade and Du (2025) proposed a deep learning-based skin cancer detection system that focuses on CNN models with data augmentation and GAN. The work attends to the problem of limited and imbalanced datasets. The study shows that augmentation strategies improve classification performance through optimized training. Nevertheless, hybrid CNN–Transformer feature learning, attention-based fusion, and efficient feature optimization are missing in this approach. Thus, it fails to capture complex lesion patterns and improve interpretability. A lightweight CNN for the correct classification of BCC, SCC, and melanoma is proposed by Bogne Tchema et al. (2025). It was found that the accuracy of CNN is about 90%, which is comparable to that of YOLOv7. Furthermore, it is less heavy on the computer as compared to YOLOv7. Regardless, the model simply relies on local CNN features, and BCC and SCC are frequently misclassified, as their visual similarity is high. In the absence of segmentation or attention mechanisms, learning to obtain global features is a bottleneck to its efficiency. With this, we draw a motivation for a hybrid, stronger framework. Ozdemir and Pacal (2025) created a hybrid deep learning framework that combined ConvNeXtV2 blocks with separable self-attention to perform efficient multiclass skin cancer classification of the ISIC 2019 dataset. It achieved an accuracy of 93.48%. The model captures local features and useful global attention with an overall compact architecture. Nevertheless, the use of segmentation-guided ROI extraction for specific lesions, explicit feature optimization mechanisms for efficient learning of features relevant to lesions, and advanced explainability techniques to identify clinically interpretable features can yield an effective framework. In 2025, Dorathi Jayaseeli et al. (2025) designed the DSC-EDLMGWO framework that makes use of SE-DenseNet for the extraction of features, LSTM, ELM, and SSDA classifier that is optimized by GWO (gray wolf optimization). Moreover, the framework provided better results when tested on the ISIC and HAM10000 datasets. The feature learning performance of the model improved well with channel attention and metaheuristic optimization. Nonetheless, it has high architectural complexity, fails to carry out hybrid CNN–Transformer global feature modeling, does not perform segment-based ROI extraction, and does not utilize explainable AI techniques. Islam et al. (2026) developed a multimodal approach based on dermoscopic images and patient information to improve diagnostic sensitivity. However, there are several important issues that need to be addressed. Tembhurne et al. (2023) conducted an ensemble of conventional deep learning and traditional machine learning techniques for skin cancer detection. Likewise, the researchers proposed a CNN-based feature extraction, while handcrafted descriptors, Contourlet Transform and Local Binary Pattern (LBP) were used for classification. As per the result, the model shows an accuracy of 93% with high recall. The model utilizes automated and manual feature extraction to enhance diagnostic effectiveness. The approach does run into higher computational complexity due to the use of handcrafted features. It cannot make use of advanced architectures, though, like CNN–Transformer hybrids, attention mechanisms, segmentation-based ROI extraction or explainable AI techniques, which limit scalability and interpretability.

Apart from accuracy, another problem with using deep learning models for skin cancer detection involves finding a compromise between adequate feature representation, computational efficiency, and explainability. Skin cancer lesions tend to vary greatly within their class while showing similarities across classes in terms of their dimensions, colors, borders, and other imaging factors. In other words, although Convolutional Neural Networks (CNNs) can efficiently extract local texture information from dermoscopic images, they perform poorly in modeling their long-range spatial relationships. On the other hand, while transformers have proven superior in understanding the global context of lesions, they are associated with additional computation costs and require extensive training data. Moreover, existing approaches employ complicated ensemble models and cannot filter out irrelevant features, rendering them unsuitable for use in clinical settings. Importantly, the problem of incorporating Explainable Artificial Intelligence (XAI) in such algorithms is still unresolved because it is vital to ensure interpretability to build trust among clinicians. Finally, class imbalance must be considered because, in the melanoma-vs.-non-melanoma endpoint used here, non-melanoma images substantially outnumber melanoma images.

In view of the above difficulties, this paper presents a Lesion-aware Hybrid CNN–Transformer Network (LHCT-Net) for melanoma-vs.-non-melanoma classification. This proposed model utilizes a U-Net segmentation mechanism to concentrate on lesion regions and eliminate the interference of background noise. The lesion-aware mask-guided augmentation-based data augmentation technique is adopted to handle imbalanced datasets by synthesizing images for the minority groups. Boundary-aware EfficientNet and Swin Transformer are used for local and global information modeling, respectively, and then the attention-guided feature integration technique is applied for improved feature representations. Furthermore, to minimize redundant features, we introduce a new block, a Lightweight Feature Optimization Block (LFOB). Grad-CAM++ is used to visualize the results for interpretability. The uniqueness of the proposed LHCT-Net is not dependent upon the merger of the CNN and Transformer architectures, but rather in the harmonious implementation of lesion-aware data augmentation, boundary-aware feature extraction, optimized feature learning, and attention-based fusion.

The main contributions of this study are summarized as follows:

- Design of a hybrid CNN–Transformer model for comprehensive local and global feature learning.
- Integration of U-Net segmentation used for improved lesion-focused analysis.
- Application of Lesion-Aware Augmentation used to effectively handle class imbalance.
- Introduction of a LFOB to refine the fused representation before classification.
- Incorporation of Grad-CAM++ to provide qualitative post-hoc visualization of model attention.

The rest of the article can be outlined as follows: the literature review is presented in Section 2, followed by a description of the methodology proposed in Section 3. Experimental results along with their analyses are discussed in Section 4, while concluding remarks have been presented in Section 5.

## 2 Related works

Recent advances in deep learning have greatly enhanced skin cancer detection from dermoscopic images; nevertheless, some issues still exist related to feature representations, interpretability, imbalance, and computation efficiency. Several approaches have been suggested by many researchers in order to overcome the abovementioned issues.

A federated learning framework based on CNN architectures, for example, ResNet50 and AlexNet, was suggested by Din et al. (2026) for decentralized melanoma detection, which achieved satisfactory classification performance on ISIC and HAM10000 datasets without jeopardizing data privacy. This approach can be considered successful due to its better results on the specified data. Nevertheless, no advanced methods of hybrid feature extraction and attention were used in order to improve the detection. An advanced deep learning approach for classification of pigmented skin lesions based on texture analysis and Gray Level Co-occurrence Matrix (GLCM) features along with hair removal based on autoencoders was proposed by Gade et al. (2026). Despite the advantages of the approach, which lie in texture-based image processing, no advanced deep architecture was implemented. Elbedoui et al. (2026) introduced an ExplainableDerm-EfficientNet architecture, combining EfficientNet with class balancing using SMOTE and explainability through LIME and SHAP methods. Even though the architecture provides better explainability and solves class imbalance issues, its limitation lies in the usage of only one CNN backbone without considering any hybrid CNN–Transformer approaches or feature fusions.

Al-Rakhami et al. (2024) proposed a federated CNN-based learning model for skin cancer detection that allows learning from multiple institutions without sharing patient data. The model, although it improves scalability and privacy, does not solve issues related to feature redundancies and computational optimizations. Muthulakshmi and Hemapriya (2023) suggested an ensemble learning approach for vascular skin lesion detection using ResNet50 and VGG16 models. Even though ensemble learning increases classification accuracy, the approach becomes computationally expensive and also lacks attention and global context modeling features. In Shaikh et al. (2024), a detailed survey on ensemble deep learning models, where the effectiveness of these ensembles in enhancing generalization and preventing overfitting was shown. Nonetheless, most ensembles do not come with an efficient computation mechanism, causing the problem to be highly computationally costly.

Dogga et al. (2026) introduced a combined Vision Transformer and Graph Neural Network with region-adaptive attention to capture the global and spatial dependencies of skin lesions. This is an effective method since it increases the interpretability of features; however, there is no attempt to tackle redundancy and efficient computation. SLDCNet, a skin lesion detection and classification system based on CNN with image preprocessing, segmentation, and transfer learning, was introduced by Varma et al. (2022). It achieved outstanding accuracy; however, there was only the use of CNN for feature extraction without any form of global dependency modeling or fusion of features. Reis et al. (2022) suggested a novel model called InSiNet, a deep CNN model for skin lesion segmentation and classification using multiple ISIC datasets. Even though this method provides an efficient model, there is no application of hybrid architecture and explainable AI methods.

Hayder and Saeed (2026) presented a review study that covers deep learning techniques such as CNNs, GANs, and Vision Transformers for skin cancer detection. The authors emphasize the role of hybrid architecture and explainability; however, they did not suggest any single model to solve all issues at once. Theerthagiri (2026) employed CNN-based algorithms with preprocessing steps for skin cancer classification. This model provides enhanced performance. Yet, the algorithm requires more advanced feature fusion techniques and interpretability steps. Islam et al. (2026) introduced a multimodal framework using patient metadata and image information, in which they achieved high sensitivity using feature fusion methods. Although this method increases the performance of the classification problem, it relies on other metadata. Divakarla et al. (2025) employed a CNN-based deep learning model to diagnose skin cancer using dermoscopic images. Though the proposed technique works effectively by extracting image features, it lacks hybrid learning and explainability.

The development of hybrid architectures and feature fusion techniques for dermoscopic image analysis has improved skin cancer detection through deep learning, as shown by recent works. Reis and Turk (2024) suggested using a fusion framework that uses transformer attention and CNN-based feature extraction to obtain local and contextual information for skin lesion classification. Their approach showed better results than individual models; however, it mainly concentrates on feature fusion and does not include segmentation-driven learning and lesion-specific augmentation that are essential to focus on clinically significant areas. Hussain et al. (2026) proposed an augmentation pipeline based on conditional GANs and an ensemble of CNN-Vision transformers for the class imbalance skin cancer datasets. Although their method is effective at improving minority class representation and classification accuracy, it is implemented through a global augmentation strategy and lacks the use of lesion masks for targeted augmentation. In addition, this study does not incorporate boundary-aware feature extraction for capturing melanoma-specific characteristics such as irregular borders. Lilhore et al. (2025) proposed SkinEHDLF, a hybrid deep learning framework that integrates several deep architectures to enhance classification performance. While the model displays strong performance through hybrid learning, it does not explicitly model lesion boundaries or structured feature interaction mechanisms. The computational complexity of these hybrid systems is also not tackled. Halawani et al. (2025) proposed a CNN-Vision Transformer fused model for early skin cancer detection to take advantage of local and global features. The effectiveness of transformer-based architecture to enhance contextualized understanding is verified in their work; however, the model lacks preprocessing based on segmentation and lesion, although it may hamper the model due to background noise.

Mateen et al. (2024) proposed the hybrid architecture with the U-Net for segmentation, Inception-ResNet for feature-extraction, and Vision Transformer for classification. Although this approach effectively integrates segmentation with hybrid learning, it does not resolve the issue of feature redundancy or computational efficiency, and lacks an explicit mechanism to refine the fused features. Moreover, the information of boundary not specific is not modeled in the classification stage. To detect skin cancer, Akinrinade and Du (2025) reviewed deep machine learning methods noting the efficacy of CNN-based models and their variants in dermoscopic image classification. The significance of deep feature extraction and model generalization across datasets is highlighted in their work. Nonetheless, the analysis primarily focuses on traditional deep learning pipelines but does not consider hybrid CNN–Transformer models, lesion-aware augmentation strategies, and computational speeding solutions.

Therefore, from the above literature, it is clear that existing approaches are either focused on using CNN for extracting local features or use complicated architecture which doesn't address issues like computational complexity and redundant features. Further, little effort has been put into developing efficient models that consider both CNN and Transformer-based hybrid architectures with feature fusion and segmentation-based learning with lightweight optimization. Review of the relevant literature shows that skin cancer detection has progressed much with the help of deep learning, but there are still some major issues unsolved. Many studies mainly utilized CNN-based architectures for feature extraction. These architectures are capable of effectively capturing local texture patterns. However, they are unable to model global contextual relationships within dermoscopic images. Although some approaches come with advanced techniques such as pre-processing, segmentation and ensemble learning, they often suffer from increased computational cost, limited feature fusion mechanism and limited interpretability. Moreover, methods involving class imbalance and explainability, such as SMOTE or SHAP, targeting some challenges are not comprehensive systems that fuse all essential aspects under a single umbrella for robust skin lesion classification. Recently proposed transformer-based or hybrid approaches improve the learning of global features. However, they often ignore problems such as feature redundancy, computational efficacy, and lesion-specific data augmentation. Recently, lightweight CNN–Transformer architectures, XAI, and computationally efficient frameworks for medical image analysis have gained prominence within studies for skin cancer classification. Tang et al. (2024) designed a lightweight skin lesion classifier, called SkinSwinViT, which utilizes the Swin Transformer to enhance generalization and computational efficiency in multiclass skin cancer classifications. Additionally, Lilhore et al. (2025) presented SkinEHDLF, a hybrid deep learning framework that employs optimized feature learning strategies to achieve accurate skin cancer classification in challenging imaging environments. Moreover, Dagnaw et al. (2024) analyzed explainable skin cancer classification using Vision Transformers and XAI approaches to show the efficacy of explainability techniques, including Grad-CAM, in improving interpretability and reliability of computer-aided skin cancer diagnoses. In addition, Shah et al. (2024) explored the role of explainable AI within an optimization-based CNN approach for skin cancer diagnosis. Furthermore, recent trends in lightweight and federated learning in medical images focus on achieving reduced computational costs without compromising accuracy (Khullar et al., 2025). Although the existing approaches have enhanced the classification accuracy of skin cancer through the utilization of lightweight CNN–Transformer models and the concept of explainable AI, few works have examined lesion-aware augmentation, boundary-aware local features, attention-driven local-global fusion, and lightweight feature refinement together in a single pipeline.

To make the experimental context of representative recent studies explicit, Table 1 records the benchmark source and nominal benchmark size reported by the source papers. These sizes describe the public collections; individual studies may apply their own exclusions, augmentation, or train/test partitions.

**Table 1:** Dataset context for representative recent studies discussed in the literature review.

Study	Dataset(s)	Nominal images	Experimental context
SkinSwinViT Tang et al. (2024)	ISIC 2018 Task 3	10,015	Seven-class skin-lesion classification using a lightweight Swin-based model; this is not the same endpoint as the present binary task.
Pacal et al. (2025)	ISIC 2019; HAM10000	25,331; 10,015	Multiclass CNN–ViT/MetaFormer evaluation on two benchmark collections; protocol differs from the present binary endpoint.
Saha et al. (2025)	ISIC 2019; HAM10000	25,331; 10,015	Knowledge-distillation experiments with teacher/student models; reported results are dataset- and phase-specific.
SkinEHDLF Lilhore et al. (2025)	ISIC 2024	401,059	Hybrid ConvNeXt/EfficientNetV2/Swin framework evaluated on the ISIC 2024 collection; not directly comparable with ISIC 2019.
Mashraqi et al. (2026)	ISIC 2019	25,331	Multiclass MobileNetV2–ResNet101–ViTE framework; reported metrics are averaged across the eight-class task rather than the present binary endpoint.

From this perspective, there seems to be a significant gap in the literature for balanced strategies that can jointly optimize local-global feature learning, lesion region mining, and computation efficiency. The methodology proposed here is set to fill this gap by integrating segmentation-enabled learning, lesion-aware augmentation, boundary-aware CNN feature extraction, attention-based fusion of CNN and Transformer representations and lightweight feature refinement, with Grad-CAM++ used only for qualitative visualization. In contrast to previous studies, which only seek to build upon singular advancements, the proposed LHCT-Net integrates these functions within one lesion-focused pipeline, with the lightweight refinement block acting only on the fused feature vector rather than pruning the backbones.

## 3 Proposed LHCT-Net: lesion-aware hybrid boundary-aware EfficientNet–Transformer classification

The proposed LHCT-Net addresses a specifically defined binary endpoint: melanoma vs. non-melanoma dermoscopic images. The negative class includes the remaining ISIC 2019 diagnostic categories and must not be interpreted as a clinically benign class. The model follows a sequential lesion-focused pipeline consisting of standardized image preprocessing, fixed U-Net lesion localization, training-only mask-guided augmentation, parallel local and global feature extraction, attention-based fusion, lightweight feature refinement, binary classification, and qualitative explanation.

Figure 1 emphasizes the separation between model development and final evaluation. Training, validation, and testing images undergo the same resizing, normalization, segmentation, and feature-extraction pathway. Only the training partition receives stochastic lesion-aware augmentation. Validation data are used for early stopping and hyperparameter selection, whereas the test partition is not used for model selection.

**Figure 1:**
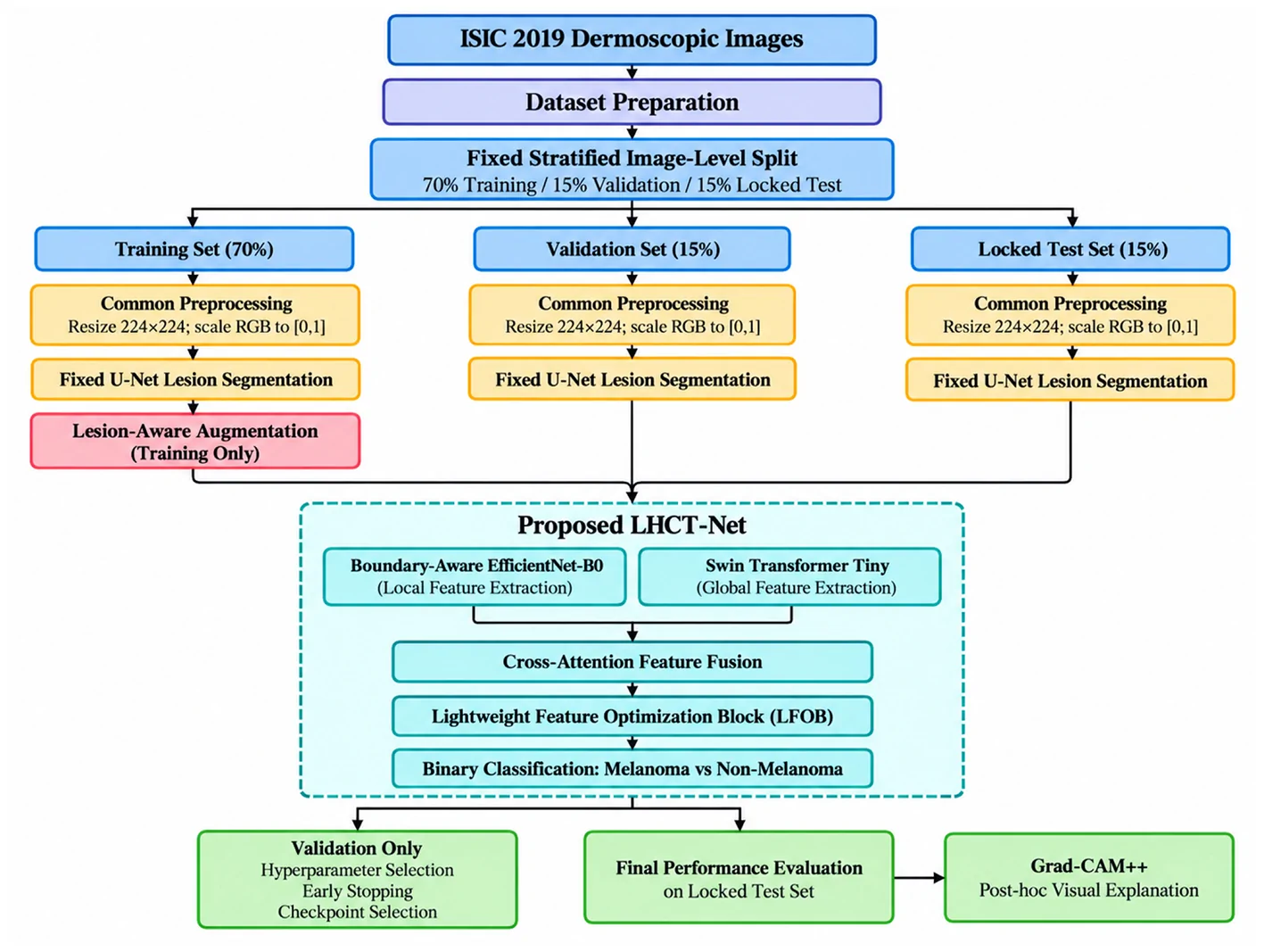
Architectural workflow of LHCT-Net for melanoma-vs.-non-melanoma classification.

### 3.1 Dataset description

The experiments use the official ISIC 2019 training collection, which contains 25,331 dermoscopic images distributed across eight diagnostic categories (International Skin Imaging Collaboration, 2019). The present study reformulates this collection as melanoma vs. non-melanoma. Melanoma images form the positive class. The negative class pools melanocytic nevus, basal cell carcinoma, actinic keratosis, benign keratosis, dermatofibroma, vascular lesion, and squamous cell carcinoma. Table 2 summarizes the melanoma and non-melanoma image counts across the training, validation and test partitions. Because basal cell carcinoma and squamous cell carcinoma are malignant and actinic keratosis is premalignant/in situ, the pooled negative class is described throughout only as *non-melanoma*; it is not described as benign or non-malignant.

**Table 2:** Distribution of the melanoma-vs.-non-melanoma endpoint used in this study.

Binary class	Total images	Training (70%)	Validation (15%)	Testing (15%)
Melanoma	4,522	3,165	678	679
Non-melanoma	20,809	14,566	3,121	3,122
**Total**	**25,331**	**17,731**	**3,799**	**3,801**

Bold values indicate the total number of images across the two classes for each partition.

The retained experiment used a fixed stratified image-level split of 70% training, 15% validation, and 15% testing. The available experiment record does not provide a complete patient identifier for every ISIC 2019 image, so a patient-wise split is not claimed. A reproducible cross-partition audit of lesion identifiers and exact/near-duplicate images was also not retained; therefore the manuscript does not claim lesion-level independence. This limitation is important because related images can otherwise introduce optimistic bias. Future validation should use lesion- or patient-grouped partitioning with an explicit duplicate audit wherever complete identifiers are available. All augmentation and parameter selection were performed after splitting and only on the training/validation data; the test set was held out for final evaluation.

The class imbalance is confined to the training procedure. Lesion-aware augmentation is applied only to melanoma images in the training set; validation and test images are never augmented. The ISIC 2019 challenge aggregates data from several sources, including HAM10000 (International Skin Imaging Collaboration, 2019). For this reason HAM10000 is not treated as an independent external validation cohort in this study.

### 3.2 Image preprocessing

RGB dermoscopic images were resized to 224 × 224 pixels. Pixel values were scaled to the [0, 1] range before entering the segmentation and classification pipeline. No normalization statistic was estimated from the validation or test partitions. The same deterministic resizing and intensity scaling were used for all three partitions, so differences between training and evaluation arose only from training-only augmentation.

### 3.3 Lesion segmentation using U-Net

A fixed U-Net is used only as a preprocessing module for lesion localization. U-Net is retained because its encoder–decoder structure and skip connections preserve fine spatial detail while providing a comparatively simple and reproducible lesion-mask generator (Ronneberger et al., 2015). More recent segmentation architectures can provide stronger boundary modeling, but the classification experiment reported here does not claim a new segmentation contribution.

For an input image $x_{i}^{norm}$, the segmentation module produces a binary mask *M*_*i*_, and the lesion-focused image is


$$x_{i}^{seg}=x_{i}^{norm}⊙M_{i}.$$

The same fixed segmentation procedure is applied independently to training, validation, and test images. The segmentation module is not fine-tuned using classification labels. Classification conclusions are therefore conditional on the quality of these generated masks. The retained source package does not include the U-Net training dataset, annotation source, checkpoint identifier, mask threshold, loss/optimizer settings, independent Dice/IoU results, or a complete audit of overlap between segmentation pretraining data and the classification partitions. These quantities are therefore not reconstructed retrospectively. The reported classification results are conditional on this fixed preprocessing stage, and the missing segmentation provenance is an explicit reproducibility limitation rather than evidence inferred from classification accuracy.

### 3.4 Lesion-aware mask-guided augmentation

Lesion-aware augmentation is restricted to the training partition. For each training sample, the same sampled geometric transform is applied to both the lesion image and its mask. Bilinear interpolation is used for RGB pixels and nearest-neighbor interpolation for masks. The transformed mask is then used during recomposition, preventing the geometric mismatch that would occur if a lesion were rotated or scaled while its support mask remained unchanged. Small boundary changes are implemented by mask dilation/erosion followed by edge feathering rather than by directly altering only lesion pixels.

The selected transformation ranges were rotation ±25°, isotropic scaling from 0.8 to 1.2, horizontal/vertical flips with probability 0.5, and mild brightness scaling within 0.9–1.1. Synthetic variants remain in the training partition and are never propagated to validation or testing. The complete training-only lesion-aware mask-guided augmentation procedure is presented in Algorithm 1.

Algorithm 1 Lesion-aware mask-guided augmentation. 
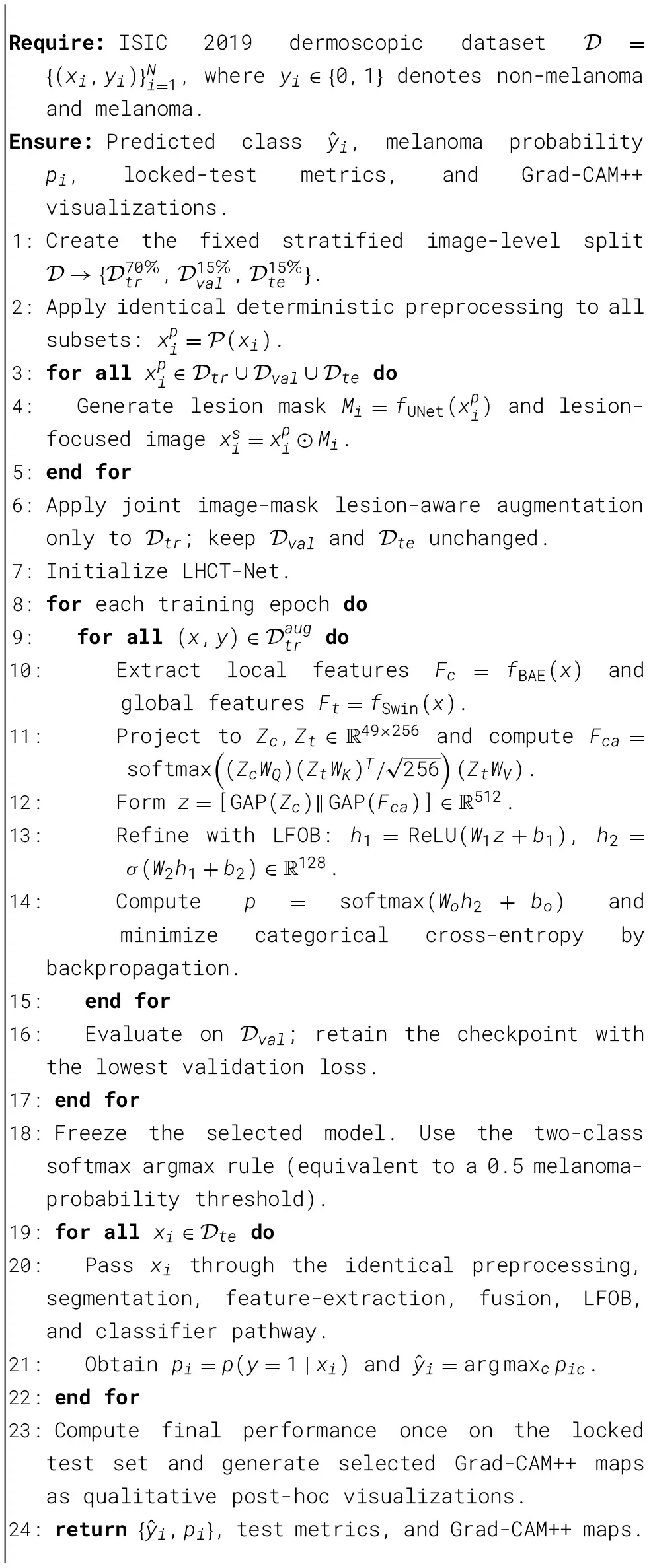

### 3.5 Lesion-aware hybrid boundary-aware EfficientNet–Transformer Network (LHCT): overview

We used the dual feature extraction method in the proposed model. The backbone CNN in use for local feature extraction is EfficientNet-B0, which is chosen for its cost-effective accuracy. The size of input images was fixed at 224 × 224 × 3. ImageNet pre-trained weights were used to initialize the EfficientNet to fine-tune during training. The local representation was based on the feature maps obtained from the last convolutional block. A standard Swin Transformer Tiny (Swin-T) backbone was used for the global feature extraction branch. The model utilized a patch size of 4, a window size of 7, and an embedding dimension of 96, followed by a hierarchical transformer with multi-head self-attention. The architecture is designed to model long-range dependencies at a manageable cost. The canonical U-Net, Swin Transformer, and Grad-CAM++ papers are retained as foundational citations because they define the methods used here; recent dermoscopy studies from 2024 to 2025 are cited in the literature review and comparative discussion to position these standard components within current skin-lesion research.

#### 3.5.1 Boundary-aware CNN for local feature extraction

To detect melanoma accurately, it heavily relies on local features like irregular borders, asymmetry and texture. While traditional CNNs sufficiently capture texture and color information, they may not well convey lesion boundaries explicitly. To overcome this limitation, it proposes a boundary-aware CNN module. Initially, a distorted or enhanced lesion image is fed into an EfficientNet backbone to extract deep local feature representations. A fixed gradient-based edge map, denoted by *E*_*i*_ = ∇*x*_*i*_, is used to represent lesion-boundary structure. A shallow CNN is applied on the edge map to extract boundary-specific features, as shown in Algorithm 2. The boundary-specific convolution is expressed in Equation 1.


$$\begin{array}{c} F_{b}=\sigma \left(W_{b}*E+b_{b}\right) \end{array}$$ (1)

Algorithm 2 Boundary-aware CNN for local lesion feature extraction. 
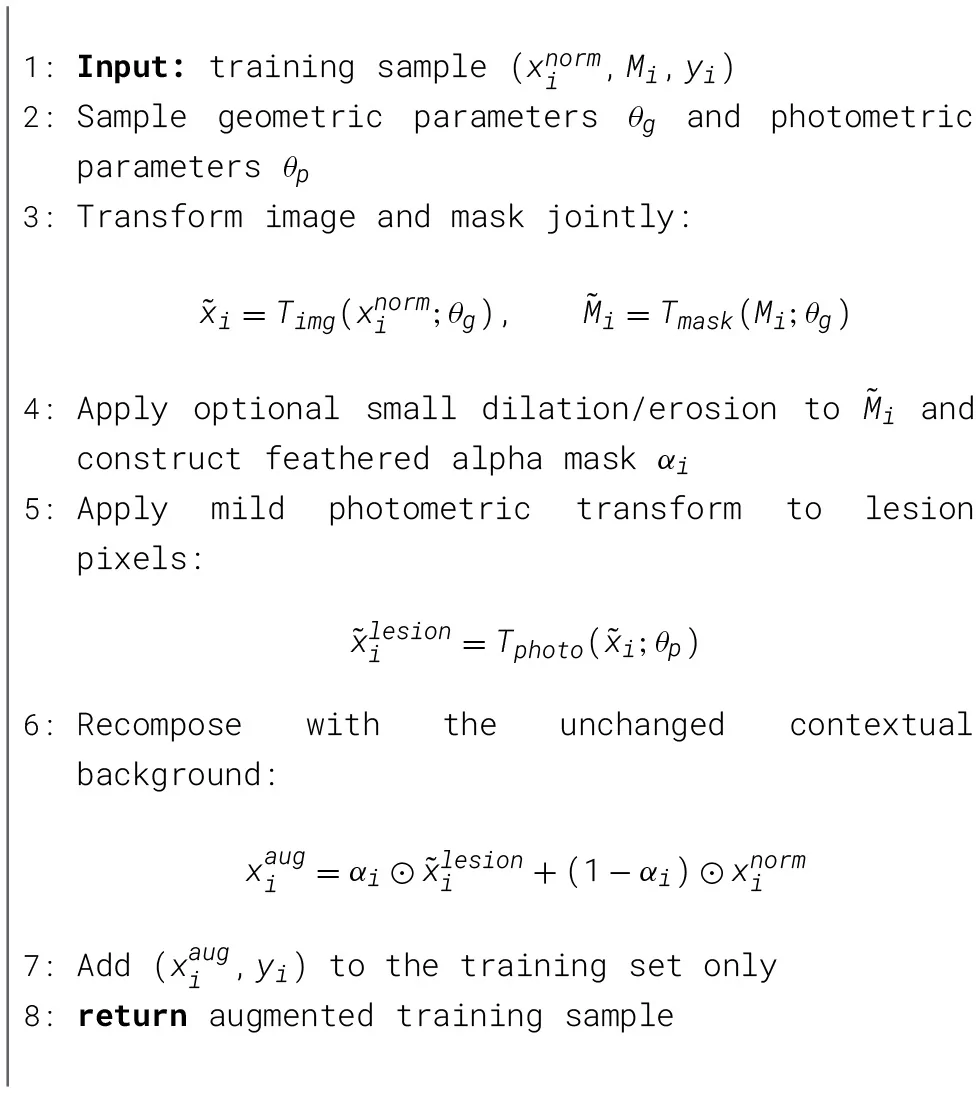

where

- *E*, boundary-enhanced feature map,
- *W*_*b*_, convolution kernel,
- *b*_*b*_, bias, and
- σ, activation function.

The Boundary-aware CNN uses convolution operations on boundary-enhanced feature maps to incorporate the contours of the lesions. In this way, the irregular border of the lesions, which is usually indicative of melanoma, is reinforced.

For an input lesion image $x_{i}\in {\mathbb{R}}^{224\times 224\times 3}$, the EfficientNet-B0 backbone extracts the deep local feature map,


$$F_{cnn}=f_{EffNet}\left(x_{i}\right)\in {\mathbb{R}}^{7\times 7\times 1280}.$$

To explicitly encode lesion-boundary information, the retained method description represents the fixed edge operation as a gradient map,


$$E_{i}=\nabla x_{i},\;E_{i}\in {\mathbb{R}}^{224\times 224\times 1}.$$

The retained implementation description records this operation only as a fixed gradient map and does not preserve the exact discrete gradient kernel or border-handling rule. The revision therefore avoids retrospectively asserting either a Sobel or Laplacian implementation; this missing low-level specification is reported as a reproducibility limitation.

Since *E*_*i*_ and *F*_*cnn*_ have different spatial and channel dimensions, the edge map is not directly added to the EfficientNet feature map. Instead, *E*_*i*_ is passed through a shallow edge-CNN with strided convolution and pooling operations to obtain:


$$F_{edge}=f_{edge}\left(E_{i}\right)\in {\mathbb{R}}^{7\times 7\times C_{e}}.$$

A 1 × 1 convolution is then used to project the edge representation to the same channel dimension as the EfficientNet feature map:


$$F_{edge}^{align}=Conv_{1\times 1}\left(F_{edge}\right)\in {\mathbb{R}}^{7\times 7\times 1280}.$$

The boundary-enhanced CNN representation is computed as:


$$F_{c}=F_{cnn}+\alpha F_{edge}^{align},$$

where α = 0.3 is the edge-weighting coefficient selected during hyperparameter tuning. This alignment ensures that element-wise addition is performed only after the spatial size and channel dimension of both feature maps are matched.

The introduced Boundary-Aware CNN specifically improves sensitivity to any irregularities in the lesion boundary, which is an important indicator of melanoma from a medical point of view. In contrast to the common CNN, whose feature extraction is mainly concerned with textures, the proposed model pays particular attention to edge-based convolutional operations that retain information about lesion boundaries.

#### 3.5.2 Swin Transformer for global feature extraction

Although CNNs work well with local features, they often fail to capture long-range dependencies and global context. Incorporating a Swin Transformer into the model alleviates this shortcoming. A hierarchical design with shifted window-based self-attention that allows for the modeling of global interactions across image regions (Liu et al., 2021). The input image is divided into patches, and hierarchical attention mechanisms are applied to learn spatial relationships across different regions of the lesion. This results in a global feature representation: Equation 2 denotes the global feature representation generated by the Swin Transformer:


$$\begin{array}{c} F_{t}=\phi_{\text{Trans}}\left(x^{seg}\right) \end{array}$$ (2)

The image of the segmented lesion was divided into patches and forwarded through a number of transformer blocks, generating a global feature representation comprising the structures and spatial relationships of the lesion. A comprehensive understanding of the lesion is established by combining this global representation with the local features extracted by a CNN.

For compatibility between the CNN and Transformer branches, both feature representations are mapped to a common token space. The boundary-enhanced CNN feature map $F_{c}\in {\mathbb{R}}^{7\times 7\times 1280}$ is first projected using a 1 × 1 convolution:


$${\tilde{F}}_{c}=Conv_{1\times 1}\left(F_{c}\right)\in {\mathbb{R}}^{7\times 7\times 256}.$$

It is then flattened into a sequence of *N* = 49 spatial tokens:


$$T_{c}=Flatten\left({\tilde{F}}_{c}\right)\in {\mathbb{R}}^{49\times 256}.$$

For the Swin Transformer branch, the input image is divided into non-overlapping 4 × 4 patches, producing an initial token grid of 56 × 56. After hierarchical patch merging, the final Swin-Tiny representation becomes:


$$F_{t}\in {\mathbb{R}}^{7\times 7\times 768}.$$

A linear projection maps the transformer output to the same embedding dimension:


$${\tilde{F}}_{t}=Linear\left(F_{t}\right)\in {\mathbb{R}}^{7\times 7\times 256},$$

and the spatial feature map is flattened as:


$$T_{t}=Flatten\left({\tilde{F}}_{t}\right)\in {\mathbb{R}}^{49\times 256}.$$

Thus, both branches provide spatially aligned token representations *T*_*c*_ and *T*_*t*_, each with 49 tokens and 256 feature dimensions, enabling attention-based local-global fusion.

Figure 2 summarizes the forward architecture for the defined melanoma-vs.-non-melanoma task. Table 3 summarizes the tensor dimensions at successive stages of the LHCT-Net forward pass. After lesion localization, only training images receive lesion-aware augmentation. Boundary-Aware EfficientNet-B0 extracts local texture and boundary information, whereas Swin Transformer Tiny provides global contextual features. The two token streams are aligned to a common 49 × 256 representation, fused by cross-attention, pooled and concatenated to a 512-dimensional vector, and refined by the LFOB to 128 dimensions before the two-class softmax classifier. The LFOB is a post-fusion refinement head and is not interpreted as pruning either backbone.

**Figure 2:**
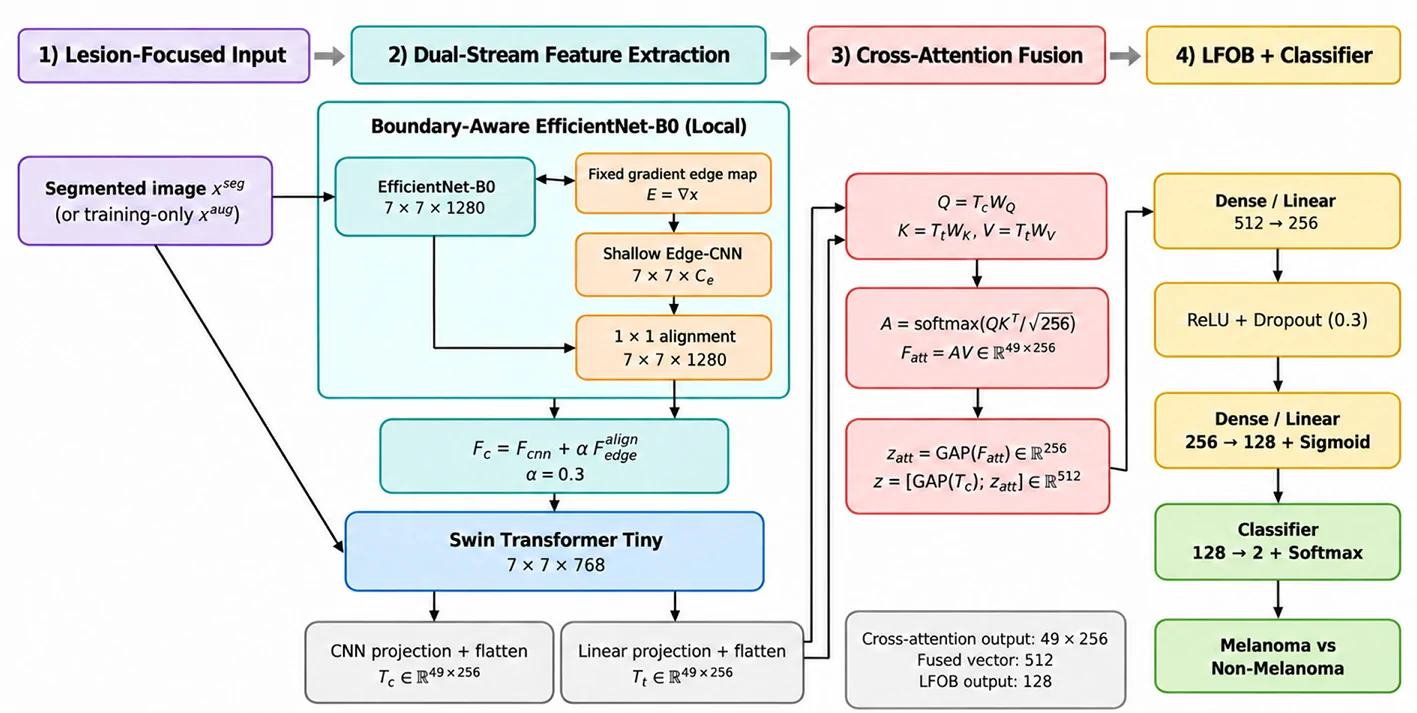
Architecture of LHCT-Net for melanoma-vs.-non-melanoma classification.

**Table 3:** Tensor dimensions in the proposed LHCT-Net forward pass.

Stage	Operation	Output dimension
Input Image	Resized dermoscopic image	224 × 224 × 3
U-Net Mask	Binary lesion mask	224 × 224 × 1
Segmented Image	$x_{i}^{seg}=x_{i}^{norm}⊙M_{i}$	224 × 224 × 3
EfficientNet-B0 Branch	Deep CNN feature map	7 × 7 × 1, 280
Edge Branch	Shallow edge-CNN output	7 × 7 × *C*_*e*_
Edge Alignment	1 × 1 convolution	7 × 7 × 1, 280
Boundary-Enhanced CNN	$F_{c}=F_{cnn}+\alpha F_{edge}^{align}$	7 × 7 × 1, 280
CNN Token Projection	1 × 1 convolution + flattening	49 × 256
Swin Transformer Output	Final Swin-Tiny feature map	7 × 7 × 768
Transformer Token Projection	Linear projection + flattening	49 × 256
Attention Matrix	$\text{Softmax}\left(\frac{QK^{T}}{\sqrt{d_{k}}}\right)$	49 × 49
Attention Output	*AV*	49 × 256
Fused Vector	[GAP(*T*_*c*_);GAP(*F*_*att*_)]	512
LFOB Hidden Layer	*W*_1_*z*+*b*_1_	256
LFOB Output	*F*_*opt*_	128
Classifier Output	Softmax	2

### 3.6 Attention-based feature fusion

An attention-based fusion mechanism is used to combine local and global features effectively. The Swin Transformer provides global feature representation, while the boundary-aware CNN generates local feature representation. Both representations are embedded into a shared feature space. To determine the relative importance of each feature component, attention weights are computed, as shown in Algorithm 3. Let $T_{c}\in {\mathbb{R}}^{49\times 256}$ denote the boundary-enhanced CNN tokens and $T_{t}\in {\mathbb{R}}^{49\times 256}$ denote the Swin Transformer tokens. The query representation is generated from the CNN tokens, while the key and value representations are generated from the Transformer tokens:


$$Q=T_{c}W_{Q},\;K=T_{t}W_{K},\;V=T_{t}W_{V},$$

Algorithm 3 Attention-based local–global feature fusion and classification. 
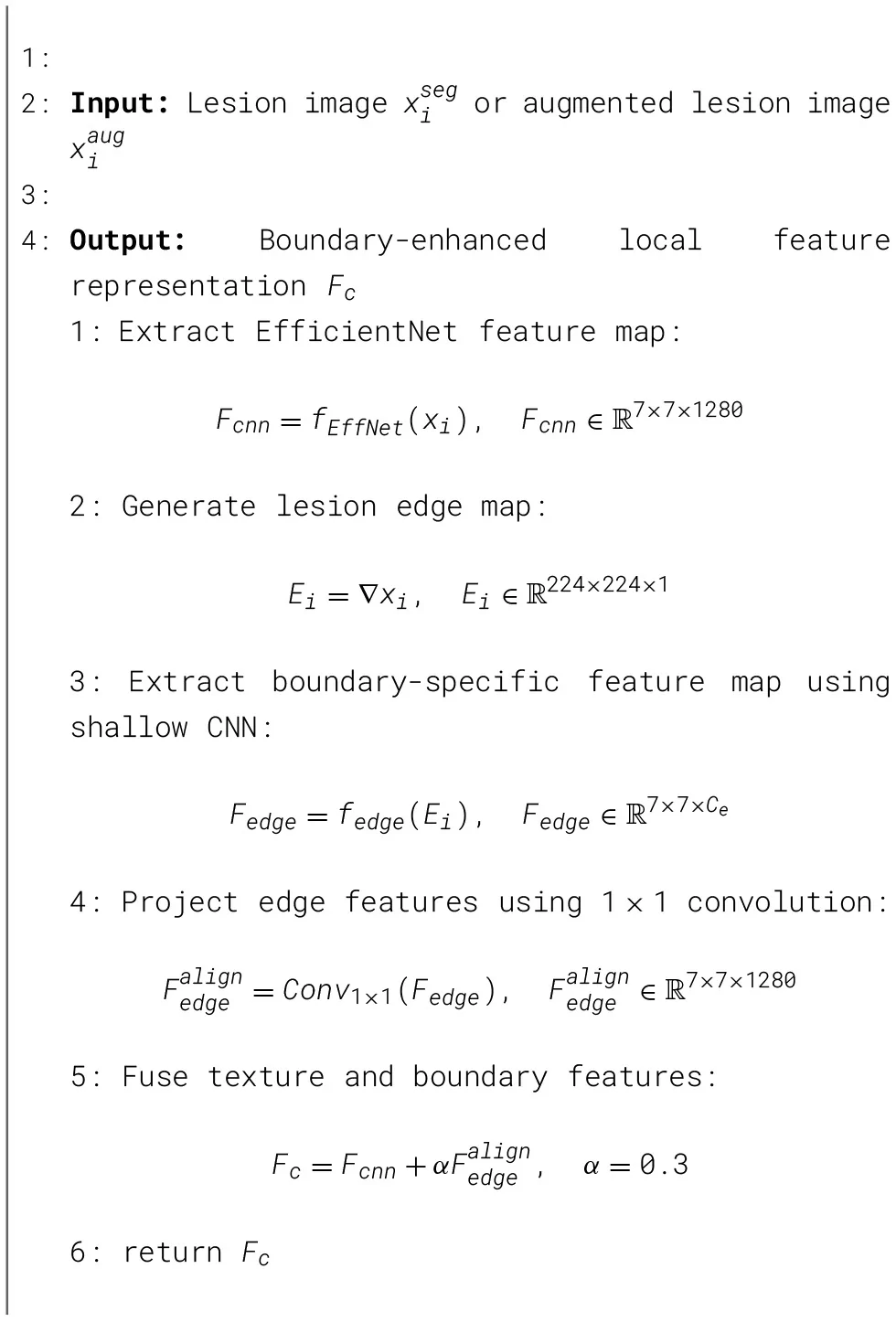

where $W_{Q},W_{K},W_{V}\in {\mathbb{R}}^{256\times 256}$. Therefore,


$$Q,K,V\in {\mathbb{R}}^{49\times 256}.$$

The scaled dot-product attention is computed as:


$$A=Softmax\left(\frac{QK^{T}}{\sqrt{d_{k}}}\right),$$

where *d*_*k*_ = 256 and *A*∈ℝ^49 × 49^. The fused local-global token representation is obtained as:


$$F_{att}=AV,\;F_{att}\in {\mathbb{R}}^{49\times 256}.$$

Global average pooling is then applied to obtain a compact fused vector:


$$z_{att}=GAP\left(F_{att}\right)\in {\mathbb{R}}^{256}.$$

To retain complementary local information, the pooled CNN representation is concatenated with the attention-fused representation:


$$z=\left[GAP\left(T_{c}\right);z_{att}\right],\;z\in {\mathbb{R}}^{512}.$$

This vector is passed to the LFOB for feature refinement.

The fusion process allows the model to enhance the most relevant features for classification methods, thus improving performance. The fused feature representation, which combines fine and coarse textural information, is proven beneficial for skin lesion classification.

### 3.7 Lightweight Feature Optimization Block (LFOB)

Fusion produces a high-dimensional representation that can contain correlated or weak activations. The LFOB is therefore implemented as a compact two-layer bottleneck head that reduces the 512-dimensional fused vector to 128 dimensions before classification. Its purpose in the present architecture is post-fusion feature refinement and dimensionality reduction rather than backbone pruning or channel-attention reweighting. Let,


$$z\in {\mathbb{R}}^{512}$$

be the fused feature vector obtained after attention-based local-global fusion. The LFOB is defined as:


$$h=\delta \left(W_{1}z+b_{1}\right),$$

$$F_{opt}=\sigma \left(W_{2}h+b_{2}\right),$$

where $W_{1}\in {\mathbb{R}}^{256\times 512}$, $b_{1}\in {\mathbb{R}}^{256}$, $W_{2}\in {\mathbb{R}}^{128\times 256}$, and $b_{2}\in {\mathbb{R}}^{128}$. Here, δ(·) denotes the ReLU activation function, and σ(·) denotes the sigmoid activation function. The first transformation reduces the fused feature dimension from 512 to 256, while the second transformation produces a compact 128-dimensional optimized feature vector:


$$F_{opt}\in {\mathbb{R}}^{128}.$$

Dropout with a rate of 0.3 is applied after the ReLU layer to reduce overfitting. The optimized representation *F*_*opt*_ is finally passed to the classifier:


$$\hat{p}=Softmax\left(W_{c}F_{opt}+b_{c}\right),$$

where $W_{c}\in {\mathbb{R}}^{2\times 128}$, $b_{c}\in {\mathbb{R}}^{2}$, and $\hat{p}\in {\mathbb{R}}^{2}$ gives the non-melanoma and melanoma probabilities. The LFOB is a compact refinement head; it does not prune or remove parameters from the EfficientNet-B0 or Swin-T backbones. With the stated dimensions, the two LFOB projections and final classifier contain 164,482 trainable weights and biases.

The LFOB is motivated by the general principle of retaining discriminative information while compressing an intermediate representation. Related works on feature recalibration and compact representations motivate this design direction (Hu et al., 2018; Wang et al., 2020; Tishby and Zaslavsky, 2015); however, the implemented LFOB itself is a two-layer bottleneck MLP-style head and is not an SE/ECA channel-attention module. The present component analysis compares the full model with and without this head, but it does not provide a parameter-matched comparison against alternative MLP, bottleneck, or channel-attention heads. Accordingly, the observed M8–M9 difference is reported as an architectural comparison rather than proof that the LFOB is uniquely optimal.

The LFOB is used as a compact post-fusion refinement head. It reduces the fused representation from 512 to 128 dimensions through two learned projections while preserving the final classification pathway. The present experiment supports a feature-dimension reduction claim only; it does not establish an end-to-end reduction in backbone parameter count, FLOPs, memory, or latency. Because the LFOB acts only after local–global fusion, its contribution is limited to compact transformation of the fused vector. Any claim of computational advantage for the complete network would require an exact implementation-level profile of total/trainable parameters, FLOPs or MACs, serialized size, memory use, and end-to-end latency; these measurements are not available in the retained experiment record and are therefore not reported.

### 3.8 Hyperparameter tuning and classification

Hyperparameters were chosen based on performance on the validation set. The input image size was fixed at 224 × 224 to match the configured EfficientNet-B0 and Swin Transformer inputs. A batch size of 32 and a learning rate of 1 × 10^−4^ resulted in stable convergence. The hyperparameter search ranges and selected settings are provided in Table 4. Table 5 summarizes the training-only lesion-aware augmentation settings, which was rotated ±25° and had a scaling factor of 0.8–1.2, resulting in realistic lesion variations that did not alter or corrupt the diagnostic structures.

**Table 4:** Hyperparameter tuning settings.

Component	Hyperparameter	Search range	Selected value
Input	Image size	224, 256	224 × 224
Optimizer	Learning rate	10^−3^, 5 × 10^−4^, 10^−4^	10^−4^
Training	Batch size	16, 32, 64	32
Training	Epochs	30, 50, 75	50
Regularization	Dropout	0.2, 0.3, 0.5	0.3
Boundary-CNN (Proposed)	Edge weight (α)	0.1, 0.2, 0.3, 0.5	0.3
Transformer	Embedding dim	96, 128	96
Fusion	Feature dimension	128, 256, 512	256
LFOB (Proposed)	Output dimension	64, 128, 256	128

**Table 5:** Training-only lesion-aware augmentation settings.

Operation	Range/ probability	Applied to
Rotation	−25° to +25°	image + mask
Scale	0.8–1.2	image + mask
Horizontal/vertical flip	*p* = 0.5	image + mask
Brightness	0.9–1.1	lesion RGB values
Boundary perturbation	small dilation/erosion	mask, then feathered recomposition

The Adam optimizer with a learning rate 1 × 10^−4^ was used to train the model. We implemented a cosine annealing learning rate scheduler to gradually reduce the learning rate. A model was trained for 50 epochs with a batch size of 32. The fully connected layer receives the optimized feature vector, followed by softmax activation to compute the class probabilities. The last prediction is taken from the class with the greatest probability, either melanoma or non-melanoma.

The edge weighting coefficient used in the boundary-aware CNN module that produces the best balance for texture and boundary features is α = 0.3. The embedding dimension of the Swin Transformer was set to 96. By keeping the value of the fusion feature dimension to 256 and the LFOB output size is 128, we reduced redundancy while retaining discriminative information to enhance classification performance. Because the classifier uses two softmax logits, Categorical cross-entropy was used as the training loss, as shown in Equation 3:


$$\begin{array}{c} L=-\underset{c\in \left\{\text{non-melanoma},\text{melanoma}\right\}}{\sum }y_{c}\log\left({\hat{p}}_{c}\right). \end{array}$$ (3)

Early stopping with a patience of eight epochs was applied using validation loss, and the checkpoint with the lowest validation loss was retained. Final class probabilities were obtained from the two-unit softmax head; the predicted class was selected by argmax, equivalent to a 0.5 probability threshold in the two-class softmax setting.

### 3.9 Explainability using Grad-CAM++

Grad-CAM++ is used as a qualitative *post-hoc* visualization method rather than as proof of clinical interpretability (Chattopadhay et al., 2018). The target activation tensor is the final convolutional feature map of the boundary-aware EfficientNet-B0 branch before global pooling. Class-specific gradients for the melanoma output are used to form the heatmap. Heatmaps are min–max normalized to [0, 1] for display and overlaid on the corresponding dermoscopic image. These visualizations indicate where the network is locally sensitive, but they do not establish causal reasoning, clinical reliability, or dermatologist agreement. No expert reader study or quantitative localization benchmark is claimed in this work.

### 3.10 Pseudocode for proposed model

The outlined approach involves a well-defined sequential workflow that allows efficient conversion of raw dermoscopy images to feature representations required for discrimination, as shown in Algorithm 4. First, the preprocessed version $x_{i}^{\text{norm}}$ of the input image *x*_*i*_ is generated to normalize the input and decrease variability in images resulting from varying lighting conditions during acquisition. The outlined approach follows a leakage-aware sequential workflow for converting raw dermoscopic images into discriminative feature representations. First, the ISIC dataset is divided by a fixed stratified image-level split into training, validation, and testing subsets using a 70:15:15 ratio. Then, the fixed U-Net segmentation module is applied separately to each subset to generate lesion masks and segmented lesion images. Lesion-aware augmentation is applied only to the training subset, while the validation and testing subsets are kept unchanged. The augmented training images are then processed through the boundary-aware CNN and Swin Transformer branches for local and global feature extraction.

Algorithm 4 Overall workflow of the proposed LHCT-Net. 
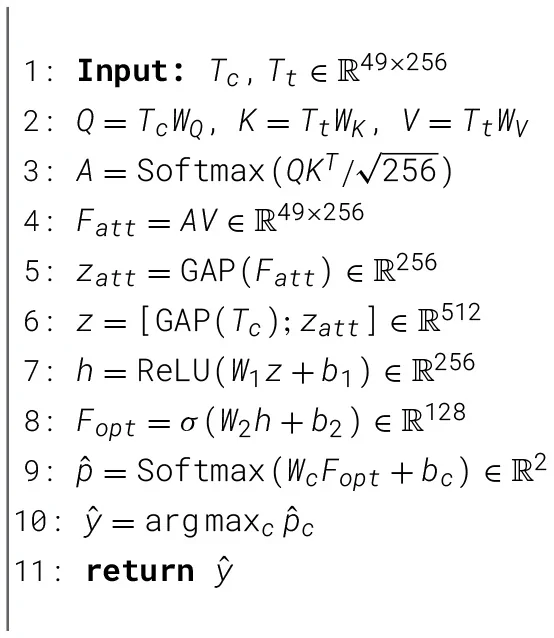

Algorithm 4 summarizes the complete workflow of the proposed LHCT-Net. Initially, the ISIC 2019 dermoscopic images are partitioned using the fixed stratified image-level 70:15:15 split described in Section 3.0.1. The same deterministic preprocessing and fixed U-Net-based lesion localization procedure are applied independently to the training, validation, and locked test partitions, whereas lesion-aware augmentation is restricted to the training partition. The resulting lesion-focused images are processed through two parallel and complementary feature-learning branches. Boundary-Aware EfficientNet-B0 extracts local texture and boundary information, while Swin Transformer Tiny captures global contextual information. The two representations are projected to the same token dimension and integrated through cross-attention-based local-global feature fusion. The fused representation is subsequently refined by the LFOB before two-class softmax classification. Hyperparameter selection, early stopping, and checkpoint selection are performed using only the validation set. The locked test set is evaluated once after model selection. Grad-CAM++ is finally applied as a qualitative post-hoc visualization method for selected test predictions.

## 4 Results and discussion

All final classification results are reported for the fixed 70:15:15 stratified image-level split described above. Augmentation and hyperparameter selection were restricted to the training and validation partitions, and the test partition was evaluated only after the final checkpoint had been selected. Because the retained experiment record does not support a fully verifiable patient-wise grouping variable, no patient-wise or cross-validation claim is made.

The proposed model was implemented using TensorFlow/Keras on an NVIDIA T4 GPU. Random seeds were fixed to 42 for Python, NumPy, and TensorFlow to make the reported holdout experiment reproducible. Baseline and component variants used the same split and training budget. Table 6 reports the retained point estimates for the same melanoma-vs.-non-melanoma endpoint.

**Table 6:** Performance comparison on the fixed ISIC 2019 melanoma-vs.-non-melanoma holdout.

Model	Accuracy (%)	Precision (%)	Sensitivity (%)	F1-score (%)	AUC
CNN (EfficientNet-B0)	92.3	91.8	90.5	91.1	0.94
Swin Transformer	93.1	92.6	91.9	92.2	0.95
CNN + Transformer (no fusion)	94.2	93.5	92.8	93.1	0.96
LHCT-Net without LFOB	95.6	95.1	94.3	94.7	0.97
**LHCT-Net**	**98.55**	**96.15**	**95.73**	**95.94**	**0.98**

Bold values identify the proposed LHCT-Net model and its reported performance.

The test-set confusion matrix contains 3,096 true negatives, 26 false positives, 29 false negatives, and 650 true positives. Figure 3 presents the confusion matrix obtained on the locked test set. These counts reproduce the reported accuracy, precision, sensitivity, and F1-score and additionally yield 99.17% specificity, 97.45% balanced accuracy, and an MCC of 0.951. The evaluation metrics derived from the locked test-set confusion matrix are summarized in Table 7. Because the negative class contains clinically heterogeneous non-melanoma diagnoses, these values quantify melanoma discrimination only; they must not be interpreted as correct benign-vs.-malignant triage for every source diagnosis.

**Figure 3:**
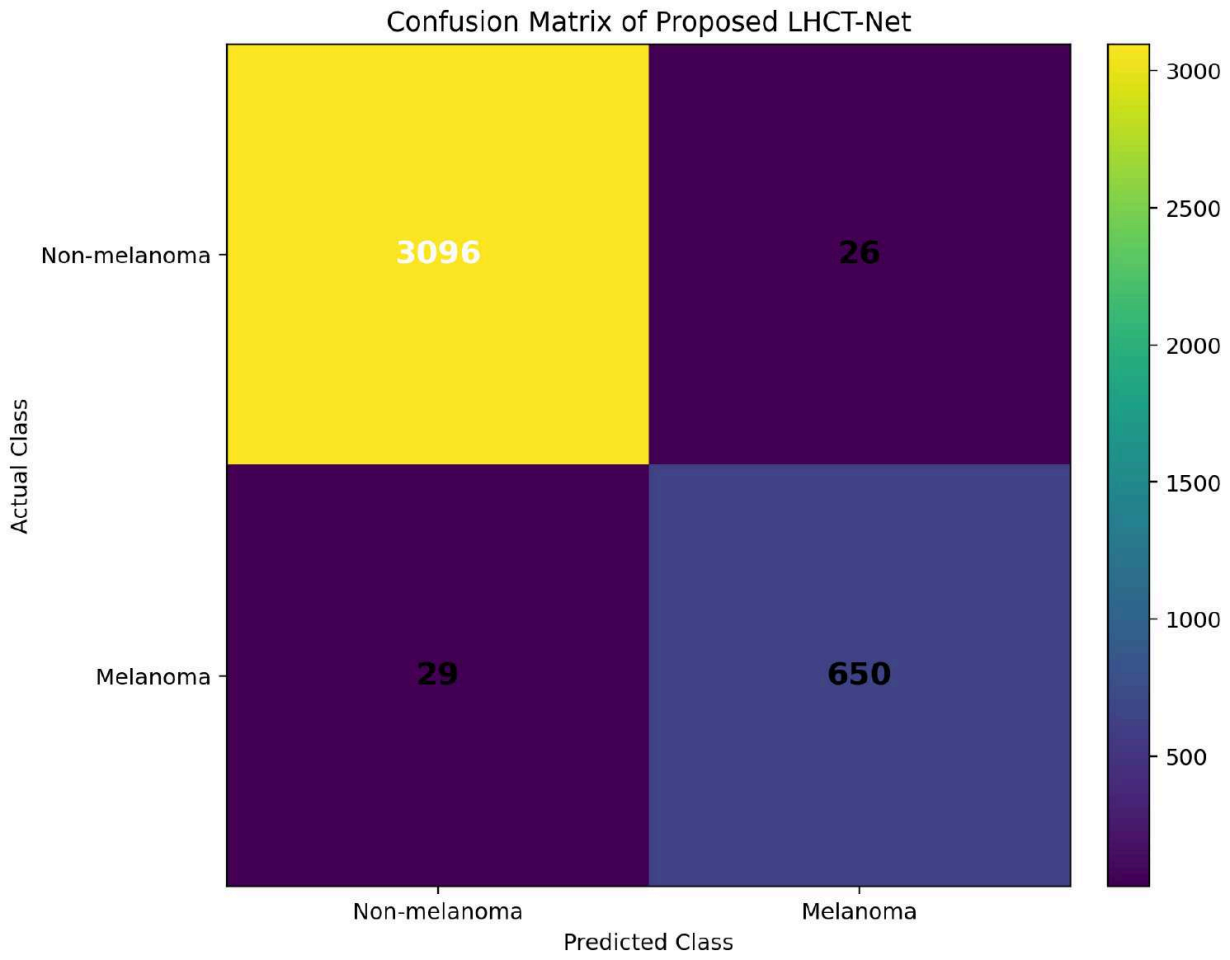
Test-set confusion matrix for melanoma vs. non-melanoma classification.

**Table 7:** Metrics derived from the locked test-set confusion matrix.

Metric	Value
Accuracy	98.55%
Melanoma precision	96.15%
Melanoma sensitivity (recall)	95.73%
Specificity	99.17%
F1-score	95.94%
Balanced accuracy	97.45%
Matthews correlation coefficient	0.951
ROC-AUC	0.980

Training and validation curves are shown only as descriptive evidence for the single reported development run and are not interpreted as cross-validation or repeated-seed uncertainty estimates Figures 4, 5 show the training and validation accuracy and loss curves, respectively, for the selected development run.

**Figure 4:**
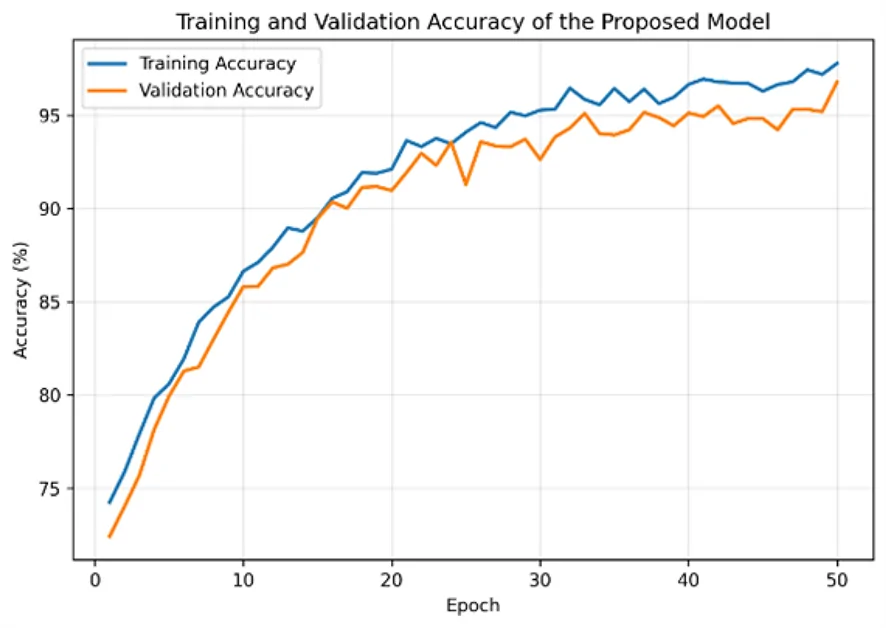
Training and validation accuracy for the selected development run.

**Figure 5:**
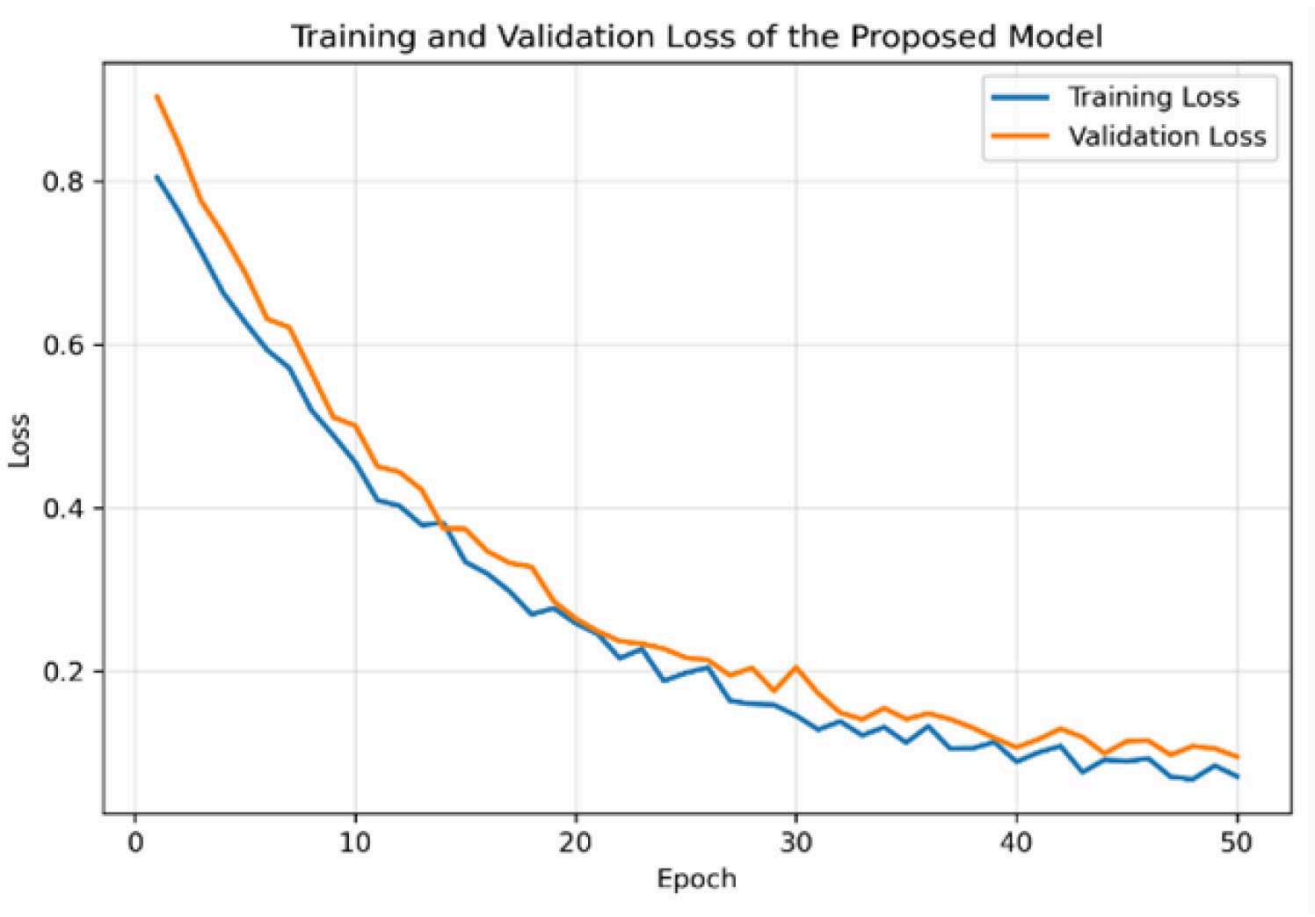
Training and validation loss for the selected development run.

### 4.1 Error analysis

For melanoma, the false-negative rate is 29/679 = 4.27%, while the false-discovery rate is 26/(650+26) = 3.85%. The false-positive rate among non-melanoma images is 26/3, 122 = 0.83%. These quantities are directly traceable to the confusion matrix rather than to repeated-seed significance testing. No paired *t*-test is used to infer statistical significance from training seeds. Case-level inference with McNemar testing, clustered bootstrap confidence intervals, AUC confidence intervals, calibration, and subgroup analysis require the underlying per-image predictions and metadata and should be included in a future confirmatory evaluation.

### 4.2 Feature contribution and interpretability scope

The component analyses are interpreted as architectural comparisons rather than clinical validation. Segmentation suppresses background regions; lesion-aware augmentation increases training diversity; the boundary-aware CNN emphasizes local lesion edges; the Transformer contributes broader spatial context; attention fusion combines the two representations; and the LFOB compresses the fused 512-dimensional representation to 128 dimensions before classification. Grad-CAM++ is retained as a qualitative visualization tool only. Expert validation, localization overlap against independent lesion/boundary annotations, faithfulness testing, and stability analysis were not performed, so claims of clinical transparency or decision-support readiness are avoided.

### 4.3 External validation scope

No independent external-validation claim is made in the present study. HAM10000 is one of the source datasets contributing to ISIC 2019 (International Skin Imaging Collaboration, 2019) and therefore cannot serve as an independent external cohort after model development on the aggregate ISIC 2019 collection unless source-level overlap is removed before training. PH^2^ is not used to support a generalization claim here because the retained experiment record does not contain the complete cohort mapping, threshold protocol, and case-level predictions needed for reproducible external evaluation.

### 4.4 Component-variant analysis

Table 8 compares retained model variants under the same endpoint and development split. The rows are not treated as a single causal, strictly nested sequence: M2–M5 probe lesion-focused CNN variants, M6 is the Transformer-only baseline, M7 assesses direct dual-backbone use without attention, M8 adds the proposed attention-based local–global fusion, and M9 adds the lightweight refinement head. The table therefore supports comparative architectural observations but does not by itself provide an isolated causal effect for every module.

**Table 8:** Component-variant analysis for the melanoma-vs.-non-melanoma task.

Variant	Model Configuration	Accuracy(%)	Precision(%)	Recall(%)	F1-score(%)	AUC
M1	CNN (EfficientNet)	92.3	91.8	90.5	91.1	0.940
M2	CNN + Segmentation	93.0	92.5	91.6	92.0	0.945
M3	CNN + Segmentation + Standard Augmentation	93.6	93.0	92.2	92.6	0.950
M4	CNN + Segmentation + Lesion-Aware Augmentation	94.4	93.8	93.1	93.4	0.955
M5	Boundary-Aware CNN	95.0	94.4	93.8	94.1	0.960
M6	Swin Transformer	93.1	92.6	91.9	92.2	0.950
M7	CNN + Transformer without Attention Fusion	94.2	93.5	92.8	93.1	0.960
M8	Proposed model without LFOB	95.6	95.1	94.3	94.7	0.970
**M9**	**Proposed model**	**98.55**	**96.15**	**95.73**	**95.94**	**0.980**

Bold values identify the complete proposed model (M9) and its reported performance.

The strongest retained point estimate is obtained by the full LHCT-Net. The large difference between M8 and M9 should be interpreted cautiously until repeated group-aware evaluation with confidence intervals is available. The present analysis is therefore reported as a controlled component comparison rather than proof that any single module independently produces the full observed gain.

### 4.5 Comparative analysis

Table 9 summarizes selected recent studies for context. Cross-paper values are descriptive because published works differ in class mapping, split policy, duplicate handling, augmentation, and evaluation protocol; they are not used as evidence of statistical superiority. A CNN-ViT based MetaFormer model with focal self-attention was proposed by Pacal et al. (2025) achieving 92.54% accuracy on ISIC 2019. While their method helps improve feature extraction through the attention mechanism, it does not explicitly add lesion-aware augmentation and boundary-aware local feature learning. In contrast, the suggested model uses segmentation-guided lesion-aware augmentation, and a boundary-aware CNN branch, which helps the model focus on clinically meaningful lesion regions and irregular misfit boundaries. Saha et al. (2025) developed a lightweight skin cancer classification model based on knowledge distillation and reported ISIC 2019 accuracies of 82.14%–84.13% in phase 1 and 81.39%–83.42% in phase 2. Their work is useful for deployment on resource-constrained devices, but the lower accuracy limits feature representation capability. The present model differs by using local-global fusion and a compact post-fusion representation; we do not claim direct efficiency superiority because profiling protocols differ.

**Table 9:** Descriptive comparison with selected recent skin-lesion classification studies; endpoints and validation protocols differ across publications.

References	Dataset	Method	Reported performance	Key limitation w.r.t. proposed model
Pacal et al. (2025)	ISIC 2019	CNN–ViT/MetaFormer with focal self-attention	Accuracy: **92.54%**; Precision: 90.41%; Recall: 87.68%; F1-score: 88.86%	Uses hybrid attention-based learning but does not include lesion-aware augmentation, boundary-aware CNN feature extraction, or LFOB-based feature optimization.
Saha et al. (2025)	ISIC 2019	Knowledge distillation-based lightweight CNN	Accuracy: **82.14%–84.13%** in Phase 1 and **81.39%–83.42%** in Phase 2	The model is lightweight and deployable, but its classification performance is lower. It does not use CNN–Transformer fusion or lesion-specific learning.
Matiray and Bhushan 2025)	ISIC 2019	Layer-wise fine-tuned ResNet models	ROC-AUC: **0.962** using ResNet50	Focuses only on fine-tuned CNN models and does not include Transformer-based global feature learning, segmentation, or lesion-aware augmentation.
Tang et al. (2024)	ISIC 2018 Task 3	SkinSwinViT: lightweight Swin Transformer-based multiclass skin lesion classifier	Accuracy: 97.88%; Precision: 97.83%; Recall: 97.55%; F1-score: 97.79%	Uses a different seven-class endpoint and ISIC 2018 protocol; therefore its headline metrics are descriptive context rather than a direct comparison with the present binary ISIC 2019 task.
Lilhore et al. (2025)	ISIC 2024	SkinEHDLF hybrid ConvNeXt–EfficientNetV2–Swin framework	Binary accuracy: 98.76%; AUROC: 99.8% (with separate multiclass results)	Uses the much larger ISIC 2024 collection and a different label formulation, so its reported performance is not directly comparable with the present ISIC 2019 endpoint.
Mashraqi et al. (2026)	ISIC 2019	MobileNetV2–ResNet101–ViTE multiclass hybrid framework	Classification accuracy: 98%; mean AUC: 96.94%	Uses an eight-class task and a different validation protocol; these values are not evidence of superiority or inferiority for the present binary endpoint.
**Proposed model**	**ISIC 2019**	**Lesion-aware boundary-enhanced CNN–Transformer with attention fusion and LFOB**	**Accuracy: 98.55%; Precision: 96.15%; Recall: 95.73%; F1-score: 95.94%; AUC: 0.980**	**–**

Boldface highlights selected reported values and the proposed model for readability; it does not imply statistical significance or direct comparability across studies.

Matiray and Bhushan (2025) applied layer-wise fine-tuning of ResNet models on ISIC 2019 and reported the best ROC AUC equal to 0.962 using ResNet50. Fine-tuned CNN models have strong discriminative ability, as shown. Nonetheless, the methodology is fundamentally reliant on CNNs and lacks modeling of global contextual dependencies via Transformer-based learning. The proposed model enhances this further by attention-based fusion of boundary-enhanced CNN local features and Swin Transformer global representations. Within the present fixed holdout experiment, LHCT-Net attains 98.55% accuracy and an AUC of 0.98. Because the compared publications use different endpoints and validation protocols, no state-of-the-art claim is made from cross-paper accuracy alone. The enhancement mainly occurs through the merging impact of lesion-aware augmentation, boundary-aware CNN feature extraction, local-global feature fusion and lightweight feature optimisation, which forms a lesion-focused framework for the defined melanoma-vs.-non-melanoma task. The internal component comparisons indicate that lesion-aware preprocessing, boundary-enhanced local features, attention-based fusion, and lightweight refinement are associated with the strongest performance in the retained experiment.

### 4.6 Discussion

The retained holdout experiment indicates that LHCT-Net can discriminate melanoma from the pooled non-melanoma category with high test-set accuracy and sensitivity. The endpoint definition is central to interpretation: BCC, SCC, and actinic keratosis are included in the negative class, so a correct “non-melanoma” prediction for those diagnoses does not mean that the lesion is clinically benign. Accordingly, the model is best described as a melanoma-vs.-non-melanoma classifier rather than a general skin-cancer detector.

The internal comparisons suggest complementary roles for local and global feature learning. EfficientNet-B0 captures local texture and morphology, whereas Swin Transformer contributes larger-scale spatial context. Attention-based fusion provides an explicit interaction between these representations, and the LFOB reduces the fused feature dimension before the final classifier. The LFOB is not claimed to reduce parameters already present in the two backbones; its role is limited to compact feature refinement.

Several limitations constrain the strength of the conclusions. First, the retained split is image-level rather than a fully verifiable patient-wise or lesion-wise partition, and a complete cross-partition duplicate/near-duplicate audit is unavailable; residual correlation between related images therefore cannot be excluded. Second, the fixed U-Net preprocessing stage lacks retained training provenance, independent segmentation benchmarking, and a complete pretraining-overlap audit. Third, the exact discrete kernel used for the recorded gradient edge operation is not preserved in the retained implementation description. Fourth, the current results do not include source-diagnosis-stratified errors, calibration, PR-AUC, subgroup performance, case-level confidence intervals, or a formal paired test based on per-image predictions. Fifth, a full layer-wise parameter/FLOP/memory/latency profile from the exact implementation is unavailable, so no end-to-end deployment-efficiency claim is made. HAM10000 is not independent of ISIC 2019 and is therefore not used as external evidence. Finally, Grad-CAM++ is qualitative and has not been validated by dermatologists or by quantitative explanation metrics. These limitations mean that the results support the technical feasibility of the defined classification pipeline, not clinical readiness.

## 5 Conclusion

This study presents LHCT-Net for the explicitly defined task of melanoma-vs.-non-melanoma classification on ISIC 2019 dermoscopic images. The framework combines fixed lesion localization, training-only mask-guided augmentation, a boundary-aware EfficientNet-B0 branch, a Swin Transformer branch, attention-based local–global fusion, a LFOB, and qualitative Grad-CAM++ visualization. On the fixed 70:15:15 image-level holdout, the test confusion matrix of 3,096 TN, 26 FP, 29 FN, and 650 TP yields 98.55% accuracy, 96.15% melanoma precision, 95.73% sensitivity, 99.17% specificity, 95.94% F1-score, 97.45% balanced accuracy, and MCC 0.951; the reported ROC-AUC is 0.98.

The results should be interpreted within the defined endpoint and experimental design. The pooled non-melanoma class contains malignant and premalignant diagnoses, patient-wise independence is not fully verifiable from the retained experiment record, and no independent external-validation or clinical-explanation claim is made. Future work should prioritize lesion/patient-grouped evaluation with explicit duplicate screening, release of split identifiers and per-image scores, subtype-wise error reporting, independent segmentation benchmarking with complete U-Net provenance, calibration and PR-AUC, clustered confidence intervals and paired case-level testing, exact model profiling, and evaluation on genuinely non-overlapping cohorts with dermatologist assessment of explanations.

## Data Availability

The original contributions presented in the study are included in the article/supplementary material, further inquiries can be directed to the corresponding author/s.
